# NASH is the Leading Cause of Hepatocellular Carcinoma in Liver Transplant Candidates

**DOI:** 10.1016/j.cgh.2023.05.019

**Published:** 2023-05-26

**Authors:** JIA HONG KOH, CHENG HAN NG, BENJAMIN NAH, DARREN JUN HAO TAN, ROHIT LOOMBA, DANIEL Q. HUANG, NASH HCC TRANSPLANT COLLABORATIVE

**Affiliations:** Division of Gastroenterology and Hepatology, Department of Medicine, National University Hospital, Singapore; Division of Gastroenterology and Hepatology, Department of Medicine, National University Hospital, Singapore; Division of Gastroenterology and Hepatology, Department of Medicine, National University Hospital, Singapore; Yong Loo Lin School of Medicine, National University of Singapore, Singapore; NAFLD Research Center, University of California at San Diego, San Diego, California; Division of Gastroenterology and Hepatology, Department of Medicine, National University Hospital, Singapore; Yong Loo Lin School of Medicine, National University of Singapore, Singapore; NAFLD Research Center, University of California at San Diego, San Diego, California

Hepatocellular carcinoma (HCC) is the third leading cause of cancer-related death globally. The burden of nonalcoholic steatohepatitis (NASH) and alcohol-related HCC has increased substantially over the last decade.^1,2^ In previous studies, NASH was determined to be the fastest-rising cause of HCC in liver transplant candidates in the United States, although chronic hepatitis C (CHC) remained the leading cause.^3,4^ However, the latest etiologic trends among waitlisted candidates in the United States are unclear. Hence, using PROCARP national registry data from the United Network for Organ Sharing database from 2000 to 2022, we conducted a comprehensive updated study to evaluate the impact of the latest trends in liver disease etiology on the prevalence and posttransplant survival of candidates with HCC on the liver transplant waiting list.^5,6^ We determined that NASH is the leading cause of HCC among waitlisted liver transplant candidates.

The detailed methods are provided in the supplementary material. The primary objective was to evaluate the temporal trends of HCC related to the various etiologies of liver disease among liver transplant candidates. The secondary objectives included the proportion of HCC related to the various causes of liver disease among liver transplant recipients, duration on the waitlist, receipt of a liver transplant, and posttransplant survival between causes of liver disease. Annual percentage changes were calculated with joinpoint regression.

From 2000 to 2022, a total of 51,721 individuals listed for liver transplantation had HCC and were included in this analysis. A total of 6457 were related to NASH, 20,972 were related to CHC, 2541 were related to chronic hepatitis B (CHB), 6030 were related to alcohol, 3936 were related to concomitant alcohol and CHC, and 11,785 had other causes of chronic liver disease (Figure 1A, Supplementary Table 1). The annual percentage change from 2000 to 2022 for NASH, CHC, CHB, alcohol, alcohol and CHC, and other causes was 29.82 (confidence interval [CI], 25.43–34.37; *P* < .01), 15.55 (CI, 13.37–17.77; *P* < .01), 20.47 (CI, 17.45–23.56; *P* < .01), 19.25 (CI, 12.68–26.21; *P* < .01), 10.05 (3.17–17.39; *P* < .01), and 12.35 (CI, 8.36–16.48; *P* < .01), respectively.

From 2000 to 2022, NASH (*P* < .01) was the fastest-growing etiology of liver disease in patients with HCC on the waitlist, followed by alcohol (*P* < .01). By contrast, the proportion of waitlisted HCC patients with CHC (*P* < .01), CHB, and alcohol with CHC (*P* < .01) declined. In 2000, 1.6% of patients with HCC on the waitlist had NASH, compared with 24.3% in 2022 (Figure 1A). NASH superseded CHC as the leading cause of liver disease among patients with HCC waitlisted for liver transplantation since 2021. Similarly, the proportion of alcohol among waitlisted patients with HCC rose substantially from 11.1% in 2000 to 12.9% in 2022. In 2022, the proportion of HCC among waitlisted candidates related to NASH, CHC, CHB, alcohol, CHC with alcohol, and other causes was 20.92%, 20.58%, 4.82%, 14.97%, 3.31%, and 35.58% in males, and 34.56%, 17.28%, 3.52%, 6.88%, 1.68%, and 36.07% in females.

Compared with NASH, alcohol was associated with a significantly longer duration on the waitlist (Model 1: *β* = 16.02; 95% CI, 5.78–26.26; *P* < .01) after adjustment for multiple confounders (Supplementary Table 2). A separate model that adjusted for tumor characteristics in addition to the other confounders used in Model 1 determined similar results (Supplementary Table 2). A total of 64.6% of candidates received liver transplantation. Compared with NASH, alcohol was associated with a lower likelihood of receiving liver transplantation, after adjusting for confounders and tumor factors (subdistribution hazard ratio, 0.90; 95% CI, 0.83–0.98; *P* = .01) (Supplementary Table 2).

From 2002 to 2022, NASH was the fastest-growing cause of liver disease among liver transplant recipients who had HCC (*P* < .01), followed by alcohol (*P* < .01). By contrast, the proportion with CHC (*P* < .01), CHB, and alcohol with CHC (*P* < .01) among transplant recipients who had HCC declined. In 2002, 1.7% of transplant recipients with HCC had NASH, compared with 28.6% in 2022 (Figure 1B). In 2020, NASH superseded CHC as the leading cause of liver disease among transplant recipients who had HCC. The proportion of patients who received a transplant for alcohol-associated HCC was 10.8% in 2002 and 19.8% in 2022.

In multivariable-adjusted analysis, CHC was associated with a greater risk of posttransplant mortality compared with NASH (Model 1: hazard ratio [HR], 1.15; 95% CI, 1.03–1.29; *P* = .01) with consistent results in a model that accounted for tumor factors (Model 2: HR, 1.13; 95% CI, 1.00–1.28; *P* = .05). Similarly, the combination of alcohol and CHC had a higher relative risk of posttransplant mortality compared with NASH (Model 1: HR, 1.27; 95% CI, 1.18–1.37; *P* < .01), which may be related to a greater risk of malignancy in this population. The higher risk of posttransplant mortality in CHC and alcohol and CHC, compared with NASH, remained consistent in subgroup analyses for sex (Supplementary Table 2). The association of liver disease etiology and graft-, infection-, and cardiovascular disease–related mortality are summarized in Supplementary Table 2.

This nationwide study provides updated data that demonstrate the impact of the changing cause of liver disease on liver transplantation. NASH has superseded CHC as the leading cause of HCC in waitlisted candidates and transplant recipients. Although liver transplantation is the ideal treatment for HCC, it is limited by the scarcity of donor organs.^7–9^ The current study highlights the enormous burden that NASH exerts on the health care system and patients by increasing the demand for donor organs and health care resources. Limitations include its retrospective nature and the lack of adjustment for obesity in the multivariable models.

To mitigate the burden of HCC, a paradigm shift in the management of patients with chronic liver disease is required. Early detection and implementation of lifestyle modifications, medical therapy, and surveillance may help reduce the burden of HCC.^10^ In this nationwide study, we determined that NASH is the leading cause of HCC among liver transplant candidates in the United States. These data highlight the urgent need to address the rapidly evolving landscape of HCC and have important implications for clinical practice and health care policy.

## Supplementary Material

s

## Figures and Tables

**Figure 1.**
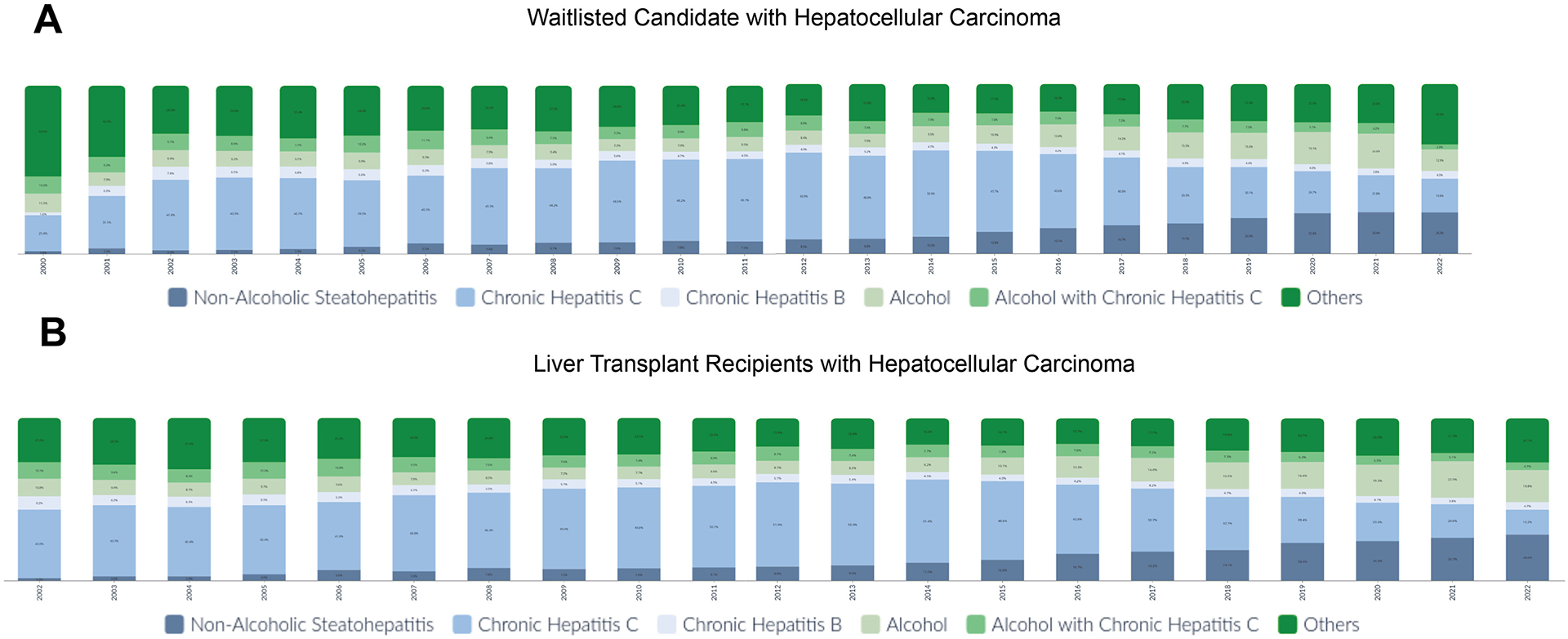
(*A*) Waitlisted candidates with hepatocellular carcinoma. (*B*) Liver transplant recipients with hepatocellular carcinoma.

## Abbreviations used in this paper:

CHB: chronic hepatitis B
CHC: chronic hepatitis C
CI: confidence interval
HCC: hepatocellular carcinoma
HR: hazard ratio
NASH: nonalcoholic steatohepatitis
