# Supplementary material for: NASH is the Leading Cause of Hepatocellular Carcinoma in Liver Transplant Candidates
Source: Clin Gastroenterol Hepatol. Author manuscript; Available in PMC 2024 Mar 14. (PMC10939135; doi:10.1016/j.cgh.2023.05.019)
Supplement: s [file NIHMS1971115-supplement-s.docx]

**Supplementary Table 1. Baseline Characteristics of Liver Transplant Candidates with Hepatocellular Carcinoma**

| Empty Cell | **NASH n = 6457** | **Chronic hepatitis C n = 20,972** | **Chronic hepatitis B n = 2541** | **Alcohol n = 6030** | **Alcohol and chronic hepatitis C n = 3936** | **Others n = 11,785** | ***P* value** |
| --- | --- | --- | --- | --- | --- | --- | --- |
| **At the time of listing** | | | | | | |  |
| **Age, *y*** | 64.00 (IQR, 60.00–68.00) | 60.00 (IQR, 55.00–64.00) | 58.00 (IQR, 52.00–65.00) | 61.00 (IQR, 55.00–66.00) | 58.00 (IQR, 53.00–62.00) | 60.00 (IQR, 54.00–65.00) | < .01 |
| **Male, *%*** | 65.62 (95% CI, 64.17–67.04) | 77.28 (95% CI, 76.58–77.97) | 84.24 (95% CI, 82.41–85.90) | 87.16 (95% CI, 86.08–88.18) | 88.37 (95% CI, 87.07–89.55) | 70.46 (95% CI, 69.39–71.51) | < .01 |
| **Race/ethnicity, *%*** |  |  |  |  |  |  |  |
| **White** | 73.10 (95% CI, 71.73–74.42) | 67.12 (95% CI, 66.34–67.90) | 22.67 (95% CI, 20.73–24.73) | 70.16 (95% CI, 68.71–71.58) | 69.95 (95% CI, 68.15–71.69) | 65.83 (95% CI, 64.72–66.92) | < .01 |
| **African American** | 1.07 (95% CI, 0.80–1.43) | 12.11 (95% CI, 11.58–12.66) | 10.23 (95% CI, 8.87–11.77) | 2.89 (95% CI, 2.41–3.46) | 8.49 (95% CI, 7.48–9.63) | 8.82 (95% CI, 8.19–9.51) | < .01 |
| **Hispanic** | 21.33 (95% CI, 20.12–22.60) | 14.97 (95% CI, 14.38–15.57) | 3.87 (95% CI, 3.04–4.90) | 23.91 (95% CI, 22.60–25.27) | 19.43 (95% CI, 17.94–21.00) | 13.85 (95% CI, 13.07 –14.67) | < .01 |
| **Asian** | 3.31 (95% CI, 2.81–3.90) | 4.54 (95% CI, 4.21–4.90) | 61.75 (95% CI, 59.40–64.04) | 1.74 (95% CI, 1.37–2.20) | 0.70 (95% CI, 0.44–1.11) | 10.26 (95% CI, 9.58–10.99) | < .01 |
| **Others** | 1.19 (95% CI, 0.90–1.57) | 1.26 (95% CI, 1.08–1.45) | 1.49 (95% CI, 1.01–2.19) | 1.30 (95% CI, 0.99–1.71) | 1.43 (95% CI, 1.04–1.97) | 1.24 (95% CI, 1.01–1.52) | < .01 |
| **BMI, *kg/m^2^*** | 32.01 (IQR, 28.43–35.86) | 27.98 (IQR, 25.06–31.59) | 25.38 (IQR, 22.96–28.12) | 28.82 (IQR, 25.71–32.39) | 28.08 (IQR, 24.97–31.65) | 27.63 (IQR, 24.52–31.46) | < .01 |
| **MELD score** | 11.00 (IQR, 9.00–15.00) | 10.00 (IQR, 8.00–13.00) | 9.00 (IQR, 7.00–12.00) | 13.00 (IQR, 10.00–17.00) | 12.00 (IQR, 9.00–15.00) | 10.00 (IQR, 8.00–14.00) | < .01 |
| **Albumin, *g/dL*** | 3.30 (IQR, 2.90–3.70) | 3.30 (IQR, 2.90–3.80) | 3.80 (IQR, 3.20–4.30) | 3.20 (IQR, 2.80–3.60) | 3.20 (IQR, 2.70–3.60) | 3.40 (IQR, 2.90–3.90) | < .01 |
| **INR** | 1.27 (IQR, 1.10–1.40) | 1.20 (IQR, 1.10–1.40) | 1.10 (IQR, 1.02–1.30) | 1.30 (IQR, 1.20–1.50) | 1.30 (IQR, 1.14–1.48) | 1.20 (IQR, 1.10–1.40) | < .01 |
| **Serum bilirubin, *mg/dL*** | 1.60 (IQR, 1.00–2.50) | 1.30 (IQR, 0.80–2.10) | 1.00 (IQR, 0.60–1.70) | 1.90 (IQR, 1.10–3.20) | 1.60 (IQR, 1.00–2.50) | 1.40 (IQR, 0.80–2.40) | < .01 |
| **Serum creatinine, *mg/dL*** | 0.89 (IQR, 0.70–1.10) | 0.86 (IQR, 0.72–1.00) | 0.90 (IQR, 0.78–1.04) | 0.90 (IQR, 0.75–1.10) | 0.90 (IQR, 0.75–1.06) | 0.88 (IQR, 0.70–1.04) | < .01 |
| **Serum sodium, *mEq/L*** | 138.00 (IQR, 135.00–140.00) | 138.00 (IQR, 136.00–140.00) | 139.00 (IQR, 137.00–141.00) | 137.00 (IQR, 134.00–140.00) | 137.00 (IQR, 135.00–140.00) | 138.00 (IQR, 136.00–140.00) | < .01 |

[BMI](https://www.sciencedirect.com/topics/medicine-and-dentistry/body-mass-index), body mass index; CI, confidence interval; INR, international normalized ratio; IQR, interquartile range; MELD, Model for End-Stage Liver Disease; NASH, nonalcoholic steatohepatitis.

**Supplementary Table 2. Impact of Liver Disease Etiology on Liver Transplant Waitlist Duration, Successful Liver Transplant, and Posttransplant Mortality**

| Empty Cell | **Model 1** | ***P* value** | **Model 2** | ***P* value** |
| --- | --- | --- | --- | --- |
| **Duration on the waitlist** | | | | |
| **Nonalcoholic steatohepatitis** | REF |  | REF |  |
| **Chronic hepatitis C** | β = -8.09 (95% CI, -23.91 to 7.73) | .32 | β = -5.91 (95% CI, -20.60 to 8.79) | .43 |
| **Chronic hepatitis B** | β = -2.80 (95% CI, -48.57 to 42.99) | .91 | β = 3.95 (95% CI, -39.59 to 47.50) | .86 |
| **Alcohol** | β = 16.02 (95% CI, 5.78 to 26.26) | **< .01**[**^a^**](https://www.sciencedirect.com/science/article/pii/S1542356523003890?via%3Dihub#tblS2fna) | β = 20.75 (95% CI, 11.70 to 29.79) | **< .01**[**^a^**](https://www.sciencedirect.com/science/article/pii/S1542356523003890?via%3Dihub#tblS2fna) |
| **Alcohol with chronic hepatitis C** | β = 8.33 (95% CI, -4.68 to 21.34) | .21 | β = 13.25 (95% CI, -0.57 to 27.07) | .06 |
| **Others** | β = -31.09 (95% CI, -58.60 to -3.58) | **.03** | β = - 31.78 (95% CI, -55.41 to -8.14) | **< .01**[**^a^**](https://www.sciencedirect.com/science/article/pii/S1542356523003890?via%3Dihub#tblS2fna) |
| **Liver transplant receipt** | | | | |
| **Nonalcoholic steatohepatitis** | REF |  | REF |  |
| **Chronic hepatitis C** | SHR, 1.02 (95% CI, 0.94 to 1.11) | .56 | SHR, 1.04 (95% CI, 0.96 to 1.12) | .35 |
| **Chronic hepatitis B** | SHR, 1.06 (95% CI, 0.93 to 1.20) | .42 | SHR, 1.07 (95% CI, 0.93 to 1.24) | .33 |
| **Alcohol** | SHR, 0.90 (95% CI, 0.82 to 0.99) | .03 | SHR, 0.90 (95% CI, 0.83 to 0.98) | **.01**[**^a^**](https://www.sciencedirect.com/science/article/pii/S1542356523003890?via%3Dihub#tblS2fna) |
| **Alcohol with chronic hepatitis C** | SHR, 0.93 (95% CI, 0.82 to 1.05) | .22 | SHR, 0.92 (95% CI, 0.82 to 1.04) | .18 |
| **Others** | SHR, 1.02 (95% CI, 0.88 to 1.19) | .79 | SHR, 1.07 (95% CI, 0.95 to 1.21) | .24 |
| **Overall posttransplant mortality** | | | | |
| **Nonalcoholic steatohepatitis** | REF |  | REF |  |
| **Chronic hepatitis C** | HR, 1.15 (95% CI, 1.03 to 1.29) | **.013**[**^a^**](https://www.sciencedirect.com/science/article/pii/S1542356523003890?via%3Dihub#tblS2fna) | HR, 1.13 (95% CI, 1.00 to 1.28) | **.05**[**^a^**](https://www.sciencedirect.com/science/article/pii/S1542356523003890?via%3Dihub#tblS2fna) |
| **Chronic hepatitis B** | HR, 0.88 (95% CI, 0.78 to 0.99) | **.039**[**^a^**](https://www.sciencedirect.com/science/article/pii/S1542356523003890?via%3Dihub#tblS2fna) | HR, 0.87 (95% CI, 0.73 to 1.04) | .12 |
| **Alcohol** | HR, 1.02 (95% CI, 0.88 to 1.18) | .81 | HR, 1.00 (95% CI, 0.86 to 1.16) | .97 |
| **Alcohol with chronic hepatitis C** | HR, 1.27 (95% CI, 1.18 to 1.37) | **< .01**[**^a^**](https://www.sciencedirect.com/science/article/pii/S1542356523003890?via%3Dihub#tblS2fna) | HR, 1.29 (95% CI, 1.18 to 1.41) | **< .01**[**^a^**](https://www.sciencedirect.com/science/article/pii/S1542356523003890?via%3Dihub#tblS2fna) |
| **Others** | HR, 1.14 (95% CI, 1.03 to 1.25) | **.01**[**^a^**](https://www.sciencedirect.com/science/article/pii/S1542356523003890?via%3Dihub#tblS2fna) | HR, 1.06 (95% CI, 0.97 to 1.16) | .23 |
| **Cardiovascular disease–related mortality** | | | | |
| **Nonalcoholic steatohepatitis** | REF |  | REF |  |
| **Chronic hepatitis C** | SHR, 0.92 (95% CI, 0.78 to 1.10) | .37 | SHR, 0.82 (95% CI, 0.64 to 1.05) | .12 |
| **Chronic hepatitis B** | SHR, 0.67 (95% CI, 0.45 to 1.00) | .05 | SHR, 0.59 (95% CI, 0.34 to 1.01) | .055 |
| **Alcohol** | SHR, 1.07 (95% CI, 0.85 to 1.36) | .57 | SHR, 0.98 (95% CI, 0.70 to 1.38) | .92 |
| **Alcohol with chronic hepatitis C** | SHR, 1.02 (95% CI, 0.89 to 1.17) | .78 | SHR, 0.93 (95% CI, 0.83 to 1.04) | .19 |
| **Others** | SHR, 0.89 (95% CI, 0.83 to 0.96) | **< .01** | SHR, 0.79 (95% CI, 0.71 to 0.89) | **< .01**[**^a^**](https://www.sciencedirect.com/science/article/pii/S1542356523003890?via%3Dihub#tblS2fna) |
| **Posttransplant graft-related mortality** | | | | |
| **Nonalcoholic steatohepatitis** | REF |  | REF |  |
| **Chronic hepatitis C** | SHR, 2.96 (95% CI, 1.31 to 2.93) | **< .01**[**^a^**](https://www.sciencedirect.com/science/article/pii/S1542356523003890?via%3Dihub#tblS2fna) | SHR, 2.00 (95% CI, 1.44 to 2.78) | **< .01**[**^a^**](https://www.sciencedirect.com/science/article/pii/S1542356523003890?via%3Dihub#tblS2fna) |
| **Chronic hepatitis B** | SHR, 1.25 (95% CI, 0.70 to 2.22) | .45 | SHR, 1.08 (95% CI, 0.63 to 1.86) | .79 |
| **Alcohol** | SHR, 1.14 (95% CI, 0.83 to 1.57) | .42 | SHR, 1.35 (95% CI, 0.93 to 1.96) | .12 |
| **Alcohol with chronic hepatitis C** | SHR, 2.19 (95% CI, 1.60 to 2.98) | **< .01**[**^a^**](https://www.sciencedirect.com/science/article/pii/S1542356523003890?via%3Dihub#tblS2fna) | SHR, 2.39 (95% CI, 1.88 to 3.03) | **< .01**[**^a^**](https://www.sciencedirect.com/science/article/pii/S1542356523003890?via%3Dihub#tblS2fna) |
| **Others** | SHR, 1.35 (95% CI, 0.97 to 1.88) | .08 | SHR, 1.40 (95% CI, 1.10 to 1.78) | **< .01**[**^a^**](https://www.sciencedirect.com/science/article/pii/S1542356523003890?via%3Dihub#tblS2fna) |
| **Infection-related mortality** | | | | |
| **Nonalcoholic steatohepatitis** | REF |  | REF |  |
| **Chronic hepatitis C** | SHR, 0.76 (95% CI, 0.60 to 0.96) | **.02**[**^a^**](https://www.sciencedirect.com/science/article/pii/S1542356523003890?via%3Dihub#tblS2fna) | SHR, 0.80 (95% CI, 0.68 to 0.93) | **< .01**[**^a^**](https://www.sciencedirect.com/science/article/pii/S1542356523003890?via%3Dihub#tblS2fna) |
| **Chronic hepatitis B** | SHR, 0.43 (95% CI, 0.32 to 0.56) | **< .01**[**^a^**](https://www.sciencedirect.com/science/article/pii/S1542356523003890?via%3Dihub#tblS2fna) | SHR, 0.45 (95% CI, 0.36 to 0.55) | **< .01**[**^a^**](https://www.sciencedirect.com/science/article/pii/S1542356523003890?via%3Dihub#tblS2fna) |
| **Alcohol** | SHR, 0.91 (95% CI, 0.64 to 1.29) | .59 | SHR, 0.95 (95% CI, 0.65 to 1.37) | .77 |
| **Alcohol with chronic hepatitis C** | SHR, 0.74 (95% CI, 0.56 to 0.96) | **.02**[**^a^**](https://www.sciencedirect.com/science/article/pii/S1542356523003890?via%3Dihub#tblS2fna) | SHR, 0.82 (95% CI, 0.69 to 0.97) | **.02**[**^a^**](https://www.sciencedirect.com/science/article/pii/S1542356523003890?via%3Dihub#tblS2fna) |
| **Others** | SHR, 0.86 (95% CI, 0.74 to 1.00) | **.05**[**^a^**](https://www.sciencedirect.com/science/article/pii/S1542356523003890?via%3Dihub#tblS2fna) | SHR, 0.87 (95% CI, 0.72 to 1.04) | .12 |
| **Malignancy-related mortality** | | | | |
| **Nonalcoholic steatohepatitis** | REF |  | REF |  |
| **Chronic hepatitis C** | SHR, 1.20 (95% CI, 1.09 to 1.31) | **< .01**[**^a^**](https://www.sciencedirect.com/science/article/pii/S1542356523003890?via%3Dihub#tblS2fna) | SHR, 1.16 (95% CI, 1.08 to 1.25) | **< .01**[**^a^**](https://www.sciencedirect.com/science/article/pii/S1542356523003890?via%3Dihub#tblS2fna) |
| **Chronic hepatitis B** | SHR, 1.29 (95% CI, 1.24 to 1.35) | **< .01**[**^a^**](https://www.sciencedirect.com/science/article/pii/S1542356523003890?via%3Dihub#tblS2fna) | SHR, 1.26 (95% CI, 1.13 to 1.39) | **< .01**[**^a^**](https://www.sciencedirect.com/science/article/pii/S1542356523003890?via%3Dihub#tblS2fna) |
| **Alcohol** | SHR, 1.09 (95% CI, 0.94 to 1.26) | .26 | SHR, 1.09 (95% CI, 1.01 to 1.17) | **.02**[**^a^**](https://www.sciencedirect.com/science/article/pii/S1542356523003890?via%3Dihub#tblS2fna) |
| **Alcohol with chronic hepatitis C** | SHR, 1.36 (95% CI, 1.09 to 1.70) | **< .01**[**^a^**](https://www.sciencedirect.com/science/article/pii/S1542356523003890?via%3Dihub#tblS2fna) | SHR, 1.35 (95% CI, 1.08 to 1.69) | **< .01**[**^a^**](https://www.sciencedirect.com/science/article/pii/S1542356523003890?via%3Dihub#tblS2fna) |
| **Others** | SHR, 1.27 (95% CI, 1.14 to 1.41) | **< .01**[**^a^**](https://www.sciencedirect.com/science/article/pii/S1542356523003890?via%3Dihub#tblS2fna) | SHR, 1.15 (95% CI, 1.09 to 1.22) | **< .01**[**^a^**](https://www.sciencedirect.com/science/article/pii/S1542356523003890?via%3Dihub#tblS2fna) |
| **Other causes of mortality** | | | | |
| **Nonalcoholic steatohepatitis** | REF |  | REF |  |
| **Chronic hepatitis C** | SHR, 1.22 (95% CI, 1.10 to 1.38) | **< .01**[**^a^**](https://www.sciencedirect.com/science/article/pii/S1542356523003890?via%3Dihub#tblS2fna) | SHR, 1.20 (95% CI, 1.09 to 1.32) | **< .01**[**^a^**](https://www.sciencedirect.com/science/article/pii/S1542356523003890?via%3Dihub#tblS2fna) |
| **Chronic hepatitis B** | SHR, 0.87 (95% CI, 0.61 to 1.23) | .43 | SHR, 0.87 (95% CI, 0.63 to 1.20) | .32 |
| **Alcohol** | SHR, 1.10 (95% CI, 0.88 to 1.37) | .40 | SHR, 1.05 (95% CI, 0.85 to 1.30) | .66 |
| **Alcohol with chronic hepatitis C** | SHR, 1.30 (95% CI, 1.17 to 1.43) | **< .01**[**^a^**](https://www.sciencedirect.com/science/article/pii/S1542356523003890?via%3Dihub#tblS2fna) | SHR, 1.28 (95% CI, 1.16 to 1.42) | **< .01**[**^a^**](https://www.sciencedirect.com/science/article/pii/S1542356523003890?via%3Dihub#tblS2fna) |
| **Others** | SHR, 1.29 (95% CI, 1.16 to 1.42) | **< .01**[**^a^**](https://www.sciencedirect.com/science/article/pii/S1542356523003890?via%3Dihub#tblS2fna) | SHR, 1.19 (95% CI, 1.09 to 1.30) | **< .01**[**^a^**](https://www.sciencedirect.com/science/article/pii/S1542356523003890?via%3Dihub#tblS2fna) |
| **Overall posttransplant mortality, subgroup analysis for males** | | | | |
| **Nonalcoholic steatohepatitis** | REF |  | REF |  |
| **Chronic hepatitis C** | HR, 1.12 (95% CI, 1.04 to 1.21) | **< .01**[**^a^**](https://www.sciencedirect.com/science/article/pii/S1542356523003890?via%3Dihub#tblS2fna) | HR, 1.07 (95% CI, 0.98 to 1.18) | **.05**[**^a^**](https://www.sciencedirect.com/science/article/pii/S1542356523003890?via%3Dihub#tblS2fna) |
| **Chronic hepatitis B** | HR, 0.86 (95% CI, 0.80 to 0.94) | **< .01**[**^a^**](https://www.sciencedirect.com/science/article/pii/S1542356523003890?via%3Dihub#tblS2fna) | HR, 0.83 (95% CI, 0.73 to 0.95) | **< .01**[**^a^**](https://www.sciencedirect.com/science/article/pii/S1542356523003890?via%3Dihub#tblS2fna) |
| **Alcohol** | HR, 0.99 (95% CI, 0.80 to 0.94) | .83 | HR, 0.95 (95% CI, 0.83 to 1.08) | .42 |
| **Alcohol with chronic hepatitis C** | HR, 1.23 (95% CI, 1.16 to 1.30) | **< .01**[**^a^**](https://www.sciencedirect.com/science/article/pii/S1542356523003890?via%3Dihub#tblS2fna) | HR, 1.22 (95% CI, 0.92 to 1.12) | **< .01**[**^a^**](https://www.sciencedirect.com/science/article/pii/S1542356523003890?via%3Dihub#tblS2fna) |
| **Others** | HR, 1.11 (95% CI, 1.00 to 1.22) | **.04**[**^a^**](https://www.sciencedirect.com/science/article/pii/S1542356523003890?via%3Dihub#tblS2fna) | HR, 1.01 (95% CI, 0.92 to 1.12) | .81 |
| **Overall posttransplant mortality, subgroup analysis for females** | | | | |
| **Nonalcoholic steatohepatitis** | REF |  | REF |  |
| **Chronic hepatitis C** | HR, 1.25 (95% CI, 1.00 to 1.57) | **.05**[**^a^**](https://www.sciencedirect.com/science/article/pii/S1542356523003890?via%3Dihub#tblS2fna) | HR, 1.30 (95% CI, 1.03 to 1.65) | **.03**[**^a^**](https://www.sciencedirect.com/science/article/pii/S1542356523003890?via%3Dihub#tblS2fna) |
| **Chronic hepatitis B** | HR, 0.95 (95% CI, 0.58 to 1.56) | .84 | HR, 0.97 (95% CI, 0.92 to 1.48) | .91 |
| **Alcohol** | HR, 1.09 (95% CI, 0.83 to 1.42) | .54 | HR, 1.17 (95% CI, 0.92 to 1.48) | .20 |
| **Alcohol with chronic hepatitis C** | HR, 1.41 (95% CI, 1.13 to 1.77) | **< .01**[**^a^**](https://www.sciencedirect.com/science/article/pii/S1542356523003890?via%3Dihub#tblS2fna) | HR, 1.50 (95% CI, 1.17 to 1.92) | **< .01**[**^a^**](https://www.sciencedirect.com/science/article/pii/S1542356523003890?via%3Dihub#tblS2fna) |
| **Others** | HR, 1.22 (95% CI, 1.08 to 1.38) | **< .01**[**^a^**](https://www.sciencedirect.com/science/article/pii/S1542356523003890?via%3Dihub#tblS2fna) | HR, 1.17 (95% CI, 1.03 to 1.33) | **.01**[**^a^**](https://www.sciencedirect.com/science/article/pii/S1542356523003890?via%3Dihub#tblS2fna) |

CI, confidence interval; HR, hazard ratio; Model 1, adjusted for age, gender, ethnicity, personal history of diabetes mellitus, and Model for End-Stage Liver Disease score; Model 2, adjusted for normalized age, gender, ethnicity, personal history of diabetes mellitus, Model for End-Stage Liver Disease score, number of tumors, tumor size, and α-fetoprotein levels; REF, reference value; SHR, subdistribution hazard ratio.

a

Bolded *P* value ≤ .05 denotes statistical significance.
